# Emergency medical technicians with impaired functional movement patterns have poorer dynamic balance and muscle flexibility: a cross-sectional study

**DOI:** 10.1186/s12873-026-01565-0

**Published:** 2026-05-09

**Authors:** Yi-Ju Tsai, Hsin-I Shih, Kai-Chia Cheng, Hsiang-Chin Hsu

**Affiliations:** 1 Department of Physical Therapy, College of Medicine, National Cheng Kung University, Tainan, Taiwan; 2 Institute of Allied Health Sciences, College of Medicine, National Cheng Kung University, Tainan, Taiwan; 3 Physical Therapy Center, National Cheng Kung University Hospital, Tainan, Taiwan; 4 Department of Emergency Medicine, College of Medicine, National Cheng Kung University, Tainan, Taiwan; 5 Department of Emergency Medicine, National Cheng Kung University Hospital, College of Medicine, National Cheng Kung University, Tainan, Taiwan; 6 Department of Public Health, College of Medicine, National Cheng Kung University, Tainan, Taiwan

**Keywords:** Postural balance, Movement, Physical fitness, Occupational injuries, Paramedics

## Abstract

**Background:**

The purpose of this study was to examine functional movement patterns and physical functions, including strength, flexibility, balance, and agility in emergency medical technicians (EMTs), and to compare physical function outcomes between EMTs with different levels of functional movement performance. We hypothesized that EMTs with poorer functional movement patterns would perform worse in physical function tests than those with better patterns.

**Methods:**

This cross-sectional study recruited 91 EMTs from the Bureau of Fire in Tainan, Taiwan. Functional movement patterns were assessed using the Functional Movement Screen (FMS™). Muscle strength and flexibility of the upper and lower extremities and core stability were assessed using a hand-held dynamometer and clinical tests. The dynamic balance ability of each leg was assessed using the Y-balance test, and agility was assessed using the agility T-test.

**Results:**

The average FMS™ score for all EMTs was 14.1, and 44 participants (48%) were classified as the higher-scoring group (FMS™ score higher than 14), and 47 (52%) were the lower-scoring group (FMS™ score 14 or less). The lower-scoring group exhibited significantly poorer fundamental movement patterns, reduced lower-body flexibility (sit-and-reach and straight-leg raise), and lower Y-balance test scores compared to the higher-scoring group. No differences were observed in upper and lower extremity muscle strength, core stability, shoulder mobility, or agility.

**Conclusion:**

The current results indicate that EMTs exhibit impaired functional movement patterns, with those with lower FMS™ scores demonstrating reduced lower body flexibility and dynamic balance compared with those with higher scores. These findings highlight the importance of movement-focused assessment and support the need for intervention strategies targeting movement quality, flexibility, and balance to promote occupational health in EMTs.

## Introduction

Emergency medical technicians (EMTs) play a critical role in life-saving care under intense pressure and limited resources. Their work often involves high-speed transport, emergency care, and shift-based schedules in unpredictable and hazardous conditions [1]. Over half report significant mental and physical fatigue during shifts, increasing their risk for both fatal and nonfatal injuries and resulting in lost workdays and reduced quality of life [2, 3].

EMTs experience injury rates nearly three times the national average [4, 5]. A systematic review of 12 studies found annual injury rates for paramedics ranging from 29.7 to 345.6 injuries per 1,000 workers, and EMTs have the highest rate of workers’ compensation claims among healthcare professionals [6]. Common injuries include sprains and strains, particularly in the lower back and upper limbs, often caused by repetitive motions, poor posture, excessive physical effort, or lifting and transferring patients and equipment [6, 7]. These injuries frequently lead to pain, reduced quality of life, and impacts on daily activities, social interactions, and career longevity [8]. Therefore, early risk identification and targeted, evidence-based interventions are crucial to protecting EMTs’ long-term health. 

The Functional Movement Screen (FMS™) assesses seven fundamental movement patterns to identify dysfunctions or performance limitations and screen for musculoskeletal injury risk [9, 10]. These tests evaluate basic locomotor, manipulative, and stabilizing movements by placing individuals in challenging positions to reveal weaknesses and imbalances. Corrective exercises are prescribed accordingly [11, 12]. Although extensively studied in athletes, firefighters, and soldiers [13–16], its application among EMTs remains underexplored. A 2017 systematic review of nine studies found that individuals with an FMS™ score ≤ 14 were 2.74 times more likely to sustain musculoskeletal injuries [17].

Despite the high musculoskeletal injury rate in EMTs, research on their physical evaluation and prevention strategies is limited. Existing studies have primarily focused on ergonomic assessments related to repetitive tasks such as lifting and bending, providing important insights into task-specific physical demands and injury mechanisms; however, these approaches may not fully capture underlying movement quality and postural control deficits that can be identified through movement pattern screening [18–20]. Unlike firefighters, whose fitness has been well documented [21, 22], EMTs have received little attention, with limited studies noting issues such as excess body weight, poor back strength, and reduced hamstring flexibility [23, 24]. To date, only one study reported a median FMS™ score of approximately 14 in emergency medical service professionals [25]. Further research is needed to explore FMS™ performance and its relationship to physical functions, including strength, flexibility, core stability, balance, and agility. Given the occupational demands of EMTs, these physical functions were selected because they are closely related to movement quality and postural control during patient handling, load carrying, and task performance in unstable or confined environments. In particular, adequate flexibility, strength, core stability, balance, and agility are essential for maintaining safe movement strategies and minimizing excessive mechanical stress on the musculoskeletal system. Other capacities, such as aerobic fitness or muscle power, while relevant to overall performance, were not assessed because the present study primarily focused on movement quality and postural control. 

Therefore, the main purposes of this study were (1) to examine the functional movement patterns of EMTs using the FMS™ and evaluate their physical functions, including strength, flexibility, core stability, balance, and agility, and (2) to compare physical function outcomes, including strength, flexibility, core stability, balance, and agility, between EMTs classified as having lower and higher functional movement performance based on the established FMS™ cutoff score. It was hypothesized that EMTs classified in the lower FMS™ score group would demonstrate poorer physical function performance compared with those in the higher FMS™ score group.

## Materials and methods

### Study design

This cross-sectional study investigated the quality of functional movement patterns and physical functions in EMTs. Testing was conducted in a laboratory at the Department of Physical Therapy of a local university. Ethical approval for this study was obtained from the Institutional Review Board of National Cheng Kung University Hospital (A-ER-105-335).

### Participants

Ninety-one EMTs were recruited from the Tainan City Bureau of Fire in Taiwan. All participants had at least 10 years of EMT experience and held the highest national EMT certification in Taiwan, (EMT-Paramedic, EMT-P), to ensure that the sample represented experienced professionals with relatively stable occupational physical demands and to reduce potential confounding effects related to early-career physical adaptation. Individuals with current pain or acute injuries affecting their duties or participation were excluded.

### Procedures

Anthropometric measurements, including height, weight, leg length, waist and hip circumferences, and waist-to-hip ratio, were recorded. Participants completed questionnaires on demographics, health, work experience, exercise habits, and musculoskeletal injury history. Musculoskeletal injuries were assessed using the Nordic Musculoskeletal Questionnaire (NMQ), which evaluates symptoms across nine body regions, including neck, shoulders, upper back, lower back, elbows, wrists/hands, hips/thighs, knees, and ankles/feet, using a diagram for localization [26, 27]. For each participant, a musculoskeletal symptom count was derived from the NMQ by summing the number of body regions in which symptoms were reported, with each of the nine regions counted once (range: 0–9). This variable was used for descriptive purposes and for group comparisons.

Participants then underwent standardized evaluations of functional movement patterns and physical functions. Functional movement patterns were assessed using the FMS™, while physical function assessments included measurements of muscle strength and flexibility (upper and lower limbs), core stability, agility, and dynamic balance. All assessments were performed by a single assessor with prior clinical experience using the FMS™ and standardized physical function testing protocols; however, formal inter- or intra-rater reliability was not assessed within the present study. A 5-minute warm-up consisting of general joint mobility exercises and light jogging was performed before all tests, and the order of physical assessments was randomized for each participant to minimize fatigue.

### Quality of functional movement patterns: FMS™

The quality of functional movement patterns was assessed using the FMS™, which consists of seven fundamental movement tests: deep squat, hurdle step, in-line lunge, shoulder mobility, active straight-leg raise, trunk stability push-up, and rotary stability. These movements are fundamental for jumping, running, and agility [28, 29]. Each is scored from 0 to 3, with a maximum total of 21. A score of 3 indicates balanced, symmetrical, and stable functional movement; 2 indicates that the movement is not optimal; 1 means the movement cannot be performed; and 0 indicates that attempting the movement causes pain. The scores below 14 indicate an increased risk of future injury [17]. A systematic review has demonstrated that the intra-rater and inter-rater reliability of the FMS™ is excellent [17].

### Physical functions

#### Muscle strength

Maximum isometric strength for the major upper and lower extremity muscles on the dominant side was assessed using a hand-held dynamometer (MicroFit3, Hoggn Health Industries Inc., Salt Lake City, UT, USA), including the shoulder flexors, shoulder abductors, elbow flexors, elbow extensors, hip flexors, hip extensors, hip abductors, knee flexors, and knee extensors. Maximal grip strength was measured using the JAMAR dynamometer. The dominant hand was identified as the one used to pick up an object, and the dominant leg was determined by which leg was used to kick a ball. All testing positions and methods followed standardized techniques for manual examination and performance [30]. Two trials were averaged and normalized to body weight.

#### Muscle flexibility

Flexibility was assessed using the shoulder circumduction, sit-and-reach, and straight-leg raise tests. The shoulder circumduction test assessed the mobility of the upper extremities and upper trunk, particularly the shoulder joints and thoracic spine. Participants raised a stick overhead with arms extended and progressively reduced the grip width as they moved it behind their body. The minimum hand distance was measured [31]. The sit-and-reach test assessed overall lower body mobility [32]. In a seated position with legs extended and heels 30 cm apart, participants reached forward with their hands overlapped for 2 s. The maximum reach distance, measured from a 25-cm baseline aligned to the fingertips, was recorded. The straight-leg raise test assessed hamstring muscle flexibility [32]. Participants lay supine on the ground, and the dominant leg, with the knee straight, was passively raised until pelvic rotation began. The maximum hip flexion angle was recorded. Two trials were averaged for analysis.

#### Core stability

Core stability was assessed using the Sorensen and double straight-leg raise tests. The Sorensen test evaluated the endurance of the back extensors. Participants lay prone with the hips, knees, and feet secured by straps. The upper trunk was unsupported at the level of the anterior superior iliac spine, and the arms were crossed over the chest. Time was recorded until the participant could no longer maintain the horizontal position [33]. The double straight-leg raise test assessed abdominal muscle endurance [34]. Participants lay supine with knees straight and hips flexed at 30 degrees. Time was recorded until they could no longer maintain the position. Only one trial was performed to prevent fatigue.

#### Agility

Agility, defined as the ability to change body position quickly while maintaining control, was assessed using the agility T-test [35]. Participants sprinted forward, moved laterally, and backpedaled around four cones in a standardized pattern. Two trials were conducted, and the fastest time was recorded.

#### Dynamic balance

Dynamic balance was assessed using the Y-Balance Test (YBT, Y-Balance Test Kit™, Perform Better, West Warwick, Rhode Island, USA). Participants balanced on one leg while reaching as far as possible with the other leg in three directions: anterior, posterolateral, and posteromedial, before returning to the starting position [36]. Trials were discarded if the balance was lost. Three valid attempts were averaged and normalized by leg length, measured from the anterior superior iliac spine to the medial malleolus. A composite score was calculated as the sum of reach distances divided by three times the leg length, multiplied by 100. Higher scores indicated better balance. In this study, composite scores of the YBT were predefined as the primary indicators of overall balance performance, whereas direction-specific reach distances were reported to provide descriptive clinical information.

### Statistical analysis

Descriptive statistics for anthropometric, demographic, and outcome measures were reported for all participants to provide an overview of functional movement patterns and physical functions among EMTs. Based on their FMS™ scores, participants were categorized into the lower-scoring (LS, ≤ 14) and higher-scoring (HS, > 14) groups. Between-group differences in functional movement patterns, physical functions, and participant characteristics were initially analyzed using independent t-tests for continuous variables and Chi-square tests for categorical variables. Since a significant between-group difference in age was observed, analyses of covariance (ANCOVA) were subsequently performed, with age included as a covariate, to compare physical function outcomes between the LS and HS groups. To quantify the magnitude of between-group differences for key outcomes, effect sizes (Cohen’s *d*) with corresponding 95% confidence intervals (CIs) were calculated. Given the exploratory nature of this study and the a priori selection of key outcomes, formal correction for multiple comparisons was not applied; instead, effect sizes with 95% CIs and age-adjusted analyses were used to support the robustness of the findings. Data were reported as means ± standard deviations unless otherwise specified. Statistical significance was set at *p* < 0.05. All statistical analyses were performed using SPSS 25.0 software (IBM Corp., Armonk, NY, USA).

## Results

### Participants

A total of 91 EMTs (89 males and 2 females) aged from 25 to 53 years were included in the analysis (Table 1). This sex distribution reflects the workforce demographics of the EMTs in the participating fire bureau during the recruitment period. The average FMS™ score across all participants was 14.01, ranging from 7 to 19. According to the NMQ questionnaire, 81 of the 91 participants (89%) reported musculoskeletal symptoms in the past year, most commonly in the lower back (65%), followed by shoulder (52%), neck (40%), knee (36%), hand/wrist (27%), hip/thigh (22%), foot/ankle (21%), upper back (21%), and elbow (10%).

**Table 1:** Demographic and anthropometric data of all EMTs

Mean (SD)	All EMTs (*N* = 91)	Higher-scoring (*n* = 44)	Lower-scoring (*n* = 47)	*p* value
Age (years)	36.12 (4.79)	37.18 (5.47)	35.17 (3.89)	0.048*
Height (cm)	173.97 (4.46)	173.50 (4.46)	174.40 (4.47)	0.337
Weight (kg)	77.71 (10.40)	75.66 (10.73)	79.63 (9.79)	0.069
Waist circumference (cm)	85.71 (9.36)	83.29 (9.42)	87.93 (9.38)	0.021*
Hip circumference (cm)	99.01 (5.93)	97.78 (6.11)	100.13 (5.59)	0.060
W/H ratio	0.86 (0.06)	0.85 (0.06)	0.88 (0.06)	0.037*
FMS™ score (/21)	14.01 (2.82)	16.36 (1.28)	11.81 (1.94)	< 0.001*
NMQ injured sites	2.96 (2.47)	2.80 (2.55)	3.11 (2.42)	0.552

*p value < 0.05 as significant

SD = Standard deviation, W/H ratio = waist–to-hip ratio

Based on the total FMS™ score, 44 participants (48%) were categorized as higher-scoring (HS, > 14), and 47 (52%) as lower-scoring (LS, ≤ 14). Each group included one female participant, and no gender differences were found between groups (*p* = 0.962). The basic characteristics of the two groups are presented in Table 1. The LS group was significantly younger (*p* = 0.048) and had greater waist circumference (*p* = 0.021) and waist-to-hip ratio (*p* = 0.037) than the HS group. As expected, the LS group had a significantly lower total FMS™ score than the HS group, with a mean difference of 4.55 (*p* < 0.001). However, the number of injured sites reported did not differ between the groups (*p* = 0.552).

### Functional movement patterns

The LS group scored significantly lower on the total FMS™ score and six of the seven subtests: deep squat, in-line lunge, shoulder mobility, active straight-leg raise, push-up, and rotary stability compared to the HS group (Table 2, all *p* < 0.05). There was also a trend of poorer performance in the hurdle step for the LS group (*p* = 0.050), though it did not reach statistical significance.

**Table 2:** Results of FMS™ of all participants, and between the higher-scoring and lower-scoring groups

Number of participants	All EMTs (*N* = 91)				Higher-scoring (*n* = 44)				Lower-scoring (*n* = 47)				*p* value
	0	1	2	3	0	1	2	3	0	1	2	3	
Deep Squat	4	4	48	35	1	0	15	28	3	4	33	7	< 0.001*
Hurdle Step	0	3	79	9	0	0	37	7	0	3	42	2	0.050
In-line Lunge	0	1	52	38	0	0	18	26	0	1	34	12	0.004*
Shoulder Mobility	18	2	17	54	1	0	8	35	17	2	9	19	< 0.001*
Active Straight-leg Raise	1	29	50	11	0	5	29	10	1	24	21	1	< 0.001*
Push-up	20	29	17	25	1	13	8	22	19	16	9	3	< 0.001*
Rotary Stability	6	7	76	2	0	1	42	1	6	6	34	1	0.016*

*p value < 0.05 as significant

### Physical functions

There were no significant differences between the groups in upper or lower extremity maximal muscle strength, including grip strength, or in core stability (Table 3). The LS group performed significantly worse on the sit-and-reach test (*p* = 0.001) and had a reduced straight-leg raise range of the left leg (*p* = 0.020) compared to the HS group. Right hamstrings flexibility was also lower, though not statistically significant (*p* = 0.117). No group differences were observed in shoulder mobility or agility performance.

**Table 3:** Results of strength, flexibility, core stability, and agility between the higher-scoring and lower-scoring groups

Mean (SD)	Higher-scoring (*n* = 44)	Lower-scoring (*n* = 47)	Mean difference (95% CI)	*p* value		
**Muscle strength (%Body weight)**						
Shoulder flexors	0.61 (0.17)	0.57 (0.14)	0.03 (-0.03, 0.10)	0.326		
Shoulder abductors	0.55 (0.14)	0.51 (0.10)	0.04 (-0.01, 0.09)	0.154		
Elbow flexors	0.77 (0.24)	0.81 (0.23)	-0.03 (-0.14, 0.06)	0.452		
Elbow extensors	0.50 (0.12)	0.49 (0.09)	0.01 (-0.03, 0.06)	0.563		
Hip flexors	0.65 (0.18)	0.64 (0.15)	0.01 (-0.06, 0.07)	0.870		
Hip extensors	0.74 (0.21)	0.68 (0.16)	0.06 (-0.01, 0.14)	0.104		
Hip abductors	0.71 (0.21)	0.69 (0.16)	0.01 (-0.06, 0.09)	0.708		
Knee flexors	0.56 (0.15)	0.55(0.13)	0.01 (-0.05, 0.07)	0.726		
Knee extensors	0.77 (0.22)	0.78 (0.21)	-0.01 (-0.10, 0.08)	0.826		
Grip strength	0.65 (0.13)	0.63 (0.12)	0.02 (-0.03, 0.07)	0.449		
**Core stability (sec)**						
Back extensors	105.43 (43.93)	96.81 (46.14)	8.62 (-10.29, 27.54)	0.368		
Abdominal muscles	76.69 (43.43)	69.81 (50.72)	6.89 (-12.93, 26.76)	0.493		
**Flexibility**						
Shoulder circumduction (cm)	53.74 (16.93)	57.09 (14.62)	-3.35 (-10.01, 3.30)	0.319		
Sit and reach test (cm)	29.74 (8.92)	23.36 (8.51)	6.39 (2.71, 10.06)	0.001*		
SLR of right leg (degree)	68.48 (20.07)	63.06 (11.79)	5.41 (-1.39, 12.22)	0.117		
SLR of left leg (degree)	68.72 (20.26)	60.28 (13.11)	8.44 (1.36, 15.53)	0.020*		
Agility T test (sec)	14.50 (1.68)	14.68 (2.04)	-0.18 (-0.97, 0.61)	0.650		

*p value < 0.05 as significant

SD = Standard deviation, SLR = straight leg raises

The results of the dynamic balance assessed using the YBT are presented in Table 4. The LS group demonstrated significantly shorter reach distances than the HS group for both legs in nearly all directions and for the composite score (all *p* < 0.05), except for the posterolateral direction of the right leg (*p* = 0.082).

**Table 4:** Results of dynamic balance between the higher-scoring and lower-scoring groups

Mean (SD)	Higher-scoring (*n* = 44)	Lower-scoring (*n* = 47)	Mean difference (95% CI)	*p* value
**Right leg**				
Anterior	0.75 (0.08)	0.70 (0.08)	0.05 (0.01, 0.08)	0.009*
Posteromedial	1.16 (0.09)	1.10 (0.12)	0.05 (0.01, 0.10)	0.015*
Posterolateral	1.17 (0.09)	1.13 (0.10)	0.04 (-0.01, 0.08)	0.082
Composite score	1.02 (0.07)	0.98 (0.09)	0.05 (0.01, 0.09)	0.008*
**Left leg**				
Anterior	0.75 (0.08)	0.70 (0.08)	0.05 (0.02, 0.09)	0.001*
Posteromedial	1.17 (0.10)	1.12 (0.12)	0.05 (0.00, 0.10)	0.032*
Posterolateral	1.20 (0.08)	1.14 (0.09)	0.07 (0.03, 0.10)	0.001*
Composite score	1.03 (0.08)	0.98 (0.08)	0.06 (0.02, 0.09)	0.001*

Given the significant between-group difference in age, additional ANCOVAs were performed with age included as a covariate. After adjustment for age, the between-group differences in lower-body flexibility (sit-and-reach and straight-leg raise of the left leg) and dynamic balance (YBT outcomes) remained statistically significant. To further quantify the magnitude of these group differences, effect sizes were calculated for the key outcomes. Moderate to large effects were observed for the sit-and-reach test (*d* = 0.73, 95% CI: 0.31 to 1.16) and left straight-leg raise (*d* = 0.50, 95% CI: 0.08 to 0.92). Similarly, YBT composite scores demonstrated moderate effect sizes for both the right (*d* = 0.49, 95% CI: 0.08 to 0.91) and left limbs (*d* = 0.63, 95% CI: 0.20 to 1.05).

## Discussion

FMS™ is a valuable and widely used tool for assessing dysfunctional movement patterns and identifying potential musculoskeletal injury risk across various populations. In this study, functional movement patterns in EMTs were evaluated using the FMS™, and differences in selected physical functions were examined between EMTs with higher and lower FMS™ performance, addressing the limited evidence specific to this occupational group. The results demonstrated that the average FMS™ score was 14.01, with 52% of the participants scoring ≤ 14, indicating a relatively high prevalence of movement dysfunction among EMTs. Those EMTs identified with higher injury risk demonstrated poorer functional movement patterns, reduced trunk flexibility, and impaired dynamic balance than those with lower injury risk, whereas no differences were observed in muscle strength, core stability, or agility.

EMTs face a significantly higher risk of occupational injury and fatality compared to other occupations [37]. Studies indicate that an FMS™ score ≤ 14 is associated with an increased risk of musculoskeletal injuries in various populations, including athletes, firefighters, and military personnel [17]. In this study, the average FMS™ score for EMTs was 14.01, aligning with a prior study of emergency medical service professionals with a median score of about 14 [25]. A 2019 meta-analysis found that FMS™ scores in tactical occupations ranged from 14.5 to 16.6 [38], while two recent studies reported average scores of 12 in Chinese police officers and firefighters [39, 40]. These differences may result from variations in age, gender, work experience, workload, physical activity, and previous injuries [40, 41]. Notably, over half of the participants (52%) in this study had an FMS™ score ≤ 14, consistent with previous findings in EMTs and firefighters [42, 25], suggesting a high prevalence of movement dysfunction within this occupational group. These findings highlight the need for screening and early intervention in the EMTs population, necessitating urgent attention to safeguard their occupational health.

EMTs with lower FMS™ scores in this study showed significantly poorer dynamic balance and lower trunk flexibility than those at lower risk of injury, while muscle strength, core stability, and agility were similar between groups. These results support previous studies linking decreased hamstring flexibility to low back pain, which was the most commonly reported musculoskeletal complaint (65%) in this study [24, 43]. Overexertion during patient handling and hazardous biomechanical tasks such as lifting or pulling heavy loads from low heights further emphasize the need for adequate flexibility in EMTs [44]. This study provided novel evidence regarding dynamic balance impairments in EMTs with poorer functional movement patterns. Loss of balance is a critical factor in injury events among emergency medical service workers, who frequently operate on uneven surfaces, navigate stairs, and work in confined or dimly lit environments [45]. The results are consistent with studies showing poorer balance in athletes and firefighter trainees with FMS™ scores ≤ 14 [46, 47]. These findings highlight the importance of dynamic balance and movement pattern evaluations for EMTs to mitigate their high risk of musculoskeletal injuries.

This study revealed that EMTs in the LS group were younger and had higher body weight and waist-to-hip ratio than those in the HS group. Consistent with previous studies, lower FMS™ scores have been associated with higher body mass index or fat mass [48]. However, the finding that younger EMTs had greater injury risk contrasts with previous studies associating lower FMS™ scores with older age [49]. This inconsistency suggests that age alone may not adequately reflect functional movement quality in occupational settings. Differences in work experience and development of movement strategies may provide a more plausible explanation. Younger or less experienced EMTs may be more frequently exposed to physically demanding tasks, such as patient lifting and transport, and may have had fewer opportunities to refine efficient movement patterns or receive targeted injury prevention training. A similar pattern has been reported by Krysak et al., who found that older professional golfers exhibited superior movement quality and balance performance than younger golfers across various skill levels [50]. Together, these findings suggest that experience-related adaptations and movement control proficiency, rather than age per se, may play a more important role in functional movement performance and injury risk among EMTs.

In the present EMTs cohort, no significant differences in muscle strength, core stability, or agility were found between groups, suggesting these physical capacities may not directly relate to injury risk as measured by FMS™ in this occupational setting. Evidence from other occupational or tactical populations, such as firefighter trainees and youth worker cohorts, has reported associations between FMS™ performance and measures of strength, trunk endurance, agility, or running speed [51, 52]. These findings indicate that, in work-related contexts, the relationship between physical capacities and functional movement quality may vary depending on task demands, training exposure, and job-specific movement requirements. Furthermore, variability across studies highlights the heterogeneous nature of occupational roles and workloads, underscoring the need for cautious interpretation. Studies conducted in athletic populations have also reported weak or absent associations between the FMS™ scores and measures of strength or athletic performance [47, 53]. However, these athlete-based findings largely reflect sport-specific performance demands and training adaptations and should be interpreted as providing a mechanistic context rather than direct evidence applicable to occupational Injury risk. Together, these inconclusive findings highlight the importance of population-specific analyses when evaluating functional movement patterns and their relationship to injury risk.

Functional movement training and corrective exercises have been shown to improve functional movement patterns and potentially reduce injury risk [11]. Unlike traditional strength training, which primarily targets maximal strength and power through single-joint loading, functional movement training emphasizes multi-joint coordination, balance between joint mobility and stability, and proper motor control under task-relevant conditions. Such training elements are often underemphasized in current training programs for the EMTs, which typically prioritize progressive resistance training. Future research is needed to evaluate whether functional movement training can improve FMS™ performance, balance, and flexibility, and whether such improvements translate into reduced injury risk in EMTs. Future research may also benefit from incorporating economic and operational indicators, such as injury-related absenteeism, workers’ compensation claims, or workforce availability, to better quantify the organizational impact of movement-focused screening and intervention programs in EMTs.

Several limitations in this study should be acknowledged. First, the cross-sectional design precludes causal inferences regarding relationships between functional movement patterns and physical function. Longitudinal and intervention studies are therefore needed to clarify causal mechanisms and intervention effects. Second, although a commonly used cutoff score (≤ 14) was applied to facilitate clinical and occupational interpretation, the ability of FMS™ scores to predict future injuries remains uncertain [25], and dichotomization may reduce statistical information. In addition, multiple unadjusted group comparisons may increase the risk of Type I error. Future studies should consider appropriate multiplicity control strategies and continuous analytical approaches to examine multiple functional outcomes and graded associations. Third, participants were recruited from a single city’s Bureau of Fire, and the study sample was predominantly male, with only 2 female participants, reflecting the workforce composition of EMTs in Taiwan. This limits the generalizability of the findings to EMTs in other regions and to female EMTs. Given known sex-related differences in physical functions such as flexibility and balance, caution is warranted when extrapolating these results beyond the studied population. Replication of these findings in broader, multi-site, and mixed-sex EMTs populations is therefore required to improve external validity. Fourth, age-adjusted analyses were performed; however, residual confounding from anthropometric factors such as waist circumference and waist-to-hip ratio cannot be fully excluded. In addition, the observed inverse association between age and FMS™ performance should be interpreted with caution. The relatively narrow age range of the study sample, together with the cross-sectional design, may contribute to a statistical artifact rather than reflecting a true age-related risk pattern. Future studies employing multivariable models, broader age distributions, or longitudinal designs are warranted to disentangle the independent effects of age, body composition, and functional movement patterns. Fifth, while the FMS™ and other assessment tools used in this study have demonstrated good reliability in previous literature, assessor training procedures and study-specific inter- or intra-rater reliability were not formally documented or evaluated. The absence of reported assessor calibration or reliability statistics may introduce potential measurement bias, and future studies should incorporate standardized training protocols and report reliability indices (e.g., intraclass correlation coefficients) to strengthen measurement validity. Sixth, muscle strength was assessed only on the dominant side, which limits the ability to examine bilateral strength asymmetries that may be relevant to functional movement quality and injury risk. Future studies should incorporate bilateral strength assessments to better evaluate asymmetry-related physical function characteristics in EMTs. In addition, the timing of assessments in relation to work shifts, fatigue, and sleep status was not controlled. Given the physically demanding and irregular work schedules of EMTs, acute fatigue or insufficient rest may have influenced their performance. Future studies should standardize testing conditions or document work-shift timing, fatigue, and sleep status to better account for their potential effects. Finally, although a formal a priori power calculation was not conducted, the age-adjusted effects observed in the present study were of moderate magnitude, supporting the adequacy of the sample size for detecting clinically meaningful group differences in balance and flexibility outcomes. Future studies should include a priori sample size justification based on expected effect sizes. Moreover, only muscle strength, flexibility, balance, and agility were assessed, leaving other important physical functions, such as aerobic fitness and muscle power, unexplored.

## Conclusion

Despite the high prevalence of musculoskeletal injuries reported among EMTs, evidence-based approaches for evaluating, managing, and preventing these injuries remain limited. The present study demonstrated that EMTs with lower functional movement scores showed poorer dynamic balance and trunk flexibility, while muscle strength and agility did not differ between groups. These findings underscore the value of movement-focused screening in occupational settings. Incorporating standardized tools such as the FMS™ and YBT into routine training, continuing education, or annual fitness evaluations may facilitate early identification of movement deficits and inform targeted preventive strategies. From an intervention perspective, the present findings suggest that training programs for EMTs may benefit from placing greater emphasis on flexibility and balance exercises, rather than focusing solely on muscle strength or agility.

## Data Availability

The datasets used and/or analyzed during the current study are available from the corresponding author on reasonable request.

## Abbreviations

EMTs: Emergency medical technicians
FMS™: Functional Movement Screen
NMQ: Nordic Musculoskeletal Questionnaire
YBT: Y-Balance Test
LS: Lower-scoring
HS: Higher-scoring
